# Micropulse Transscleral Cyclophotocoagulation Results in Secondary Glaucoma

**DOI:** 10.3390/life13051149

**Published:** 2023-05-09

**Authors:** Zsuzsa Szilagyi, Kinga Kranitz, Zoltan Zsolt Nagy, Zsuzsa Recsan

**Affiliations:** Department of Ophthalmology, Semmelweis University, H-1085 Budapest, Hungary

**Keywords:** micropulse cyclophotocoagulation, secondary glaucoma, silicon oil

## Abstract

The aim of this study was to analyze the long-term outcome of first session of micropulse transscleral cyclophotocoagulation (MP-CPC) for refractory glaucoma developed after vitreoretinal surgery combined with silicone oil implantation. The inclusion criteria of this consecutive case series were: patients with secondary glaucoma in the refractory stage who underwent MP-CPC between 2018 and 2021, vitreoretinal surgery combined with silicon oil implantation, and at least a 24-month follow-up period after MP-CPC. Success was defined as the baseline eye pressure reduced at least 20%, and it should be ranged between 10 to 20 mmHg without further MP-CPC at the end of the follow-up. For this retrospective study, 11 eyes of 11 patients were selected. The reduction in IOP was found to be significant (*p* = 0.004) at the end of the follow-up time, and the success rate was 72% according to our results. The change in the number of antiglaucoma agents in the administered eyedrops was not significant compared to the baseline values. At the end of the follow-up period the change in BCVA values was not significant (*p* = 0.655). Our results confirm significant IOP lowering effect of this subthreshold method preserving visual performance safely even in eyes with previous vitrectomy surgery with a silicone oil implantation.

## 1. Introduction

Glaucoma is a disease that leads to the progressive apoptosis of retinal ganglion cells, causing irreversible damage to the retinal nerve fiber layer and the optic nerve head. Secondary glaucoma is a heterogenous group of different conditions, such as uveitis, neovascularization in proliferative diabetic retinopathy or central retinal vein occlusion (CRVO), and iatrogenic causes. Iatrogenic secondary open-angle glaucoma includes cases in which a substance (e.g., silicon oil) used in the surgical treatment of a pre-existing disease (e.g., retinal detachment) leads to higher intraocular pressure. The term refractory glaucoma is usually used in cases where the intraocular pressure is uncontrollably high despite maximum eyedrop therapy or when surgical methods (e.g., cyclodestructive procedures or filtration surgery) fail to reduce the intraocular pressure.

Silicon oil implanted in the vitreous cavity can cause an increase in intraocular pressure in several ways: pupillary block glaucoma in aphakic eyes, overfilling of silicon oil, silicon oil migrating into the anterior chamber, and secondary chronic open or closed angle glaucoma [1]. The migration of emulsified silicon oil into the anterior chamber may cause trabecular meshwork occlusion and early postoperative intraocular pressure (IOP) elevation. Macrophages of the trabecular meshwork can phagocyte the silicon oil globules, and they may cause trabeculitis, which manifests as a late IOP elevation. In the long term, silicon oil can cause major structural changes in the trabecular meshwork, which may later become permanent. Risk factors for IOP elevation following pars plana vitrectomy (PPV) combined with silicon oil implantation include preoperative intraocular hypertension, high myopia, pseudophakia, and the use of low viscosity silicon oil during surgery [2,3]. Management is difficult because, apart from topical or systemic IOP decreasing drugs, trabeculectomy is not an option, as this would lead the emulsified silicone even deeper into the orbit. In addition, cyclodestructive procedures have a number of side effects, such as hypotony, phthisis, hyphaema, choroidal detachment, uveitis, macular edema, severe orbital pain, lens subluxation, further IOP elevation, and loss of vision. Traditional Neodymium: Yttrium–Aluminum–Garnet (Nd:YAG) laser or diode laser transscleral cyclophotocoagulation (CPC) works with continuous laser light emission (continuous wave CPC or CW-CPC), and it is absorbed by melanin in the ciliary epithelial cells. Cyclodestructive procedures reduce the production of aqueous humor by partially destroying the ciliary epithelial cells, thus reducing IOP. This method causes coagulation necrosis and ischemia by focused energy associated with high heat formation in the area. The problem with this method is that the power is difficult to control, and postoperative complications are common. In contrast, a micropulse laser CPC (MP-CPC) causes cold ablation without necrosis. The mechanism of action has been unknown yet. It decreases aqueous humor production and modulates the formation of inflammatory mediators, thus reducing IOP and potential fibrosis [4,5]. With this new method, the energy is divided into repetitive segments, so-called micropulses. In “on” mode, the micropulse lasts 30–300 s, followed by a “pause” of 1700–2000 s in “off” mode. A significant part of the thermal energy emitted by the 810 nm diode laser is absorbed in the pigment epithelium. A gradual release leads to protein denaturation in the cells and stress-induced apoptosis. During this time, in “off” mode, the surrounding non-pigmented tissues have time to cool down and may remain below the coagulation threshold. Recent studies found that in human cadaver eyes, MP-CPC caused less tissue disruption to the ciliary body compared with traditional and low burn CW-CPC treatments [6,7]. 

In recent years, we have seen patients who have undergone silicone oil implantation for a more complicated vitreoretinal problem and developed difficult-to-treat secondary glaucoma. Despite maximum conservative treatment, the patient may experience pain in the eye or still loses vision after a successful vitreoretinal procedure due to high intraocular pressure. However, while MP-CPC is a well-documented effective adjuvant treatment method for several types of glaucoma (see Table 1), very little data are available in cases with secondary glaucoma of a complex pathomechanism. The possible complications described in previous studies are listed in Table 2 (for details see Table A1).

The aim of this preliminary study was to analyze the long-term outcome of MP-CPC for refractory glaucoma developed after vitreoretinal surgery combined silicone oil implantation for complicated posterior segment disease.

## 2. Materials and Methods

It is a retrospective analysis of a consecutive case series. Medical records of 11 patients were analyzed; the data were extracted from Medsol system. Institutional Review Board approval was obtained for all study protocols (SE RKEB 193/2022). The inclusion criteria of this consecutive case series were: (1) patients who underwent MP-CPC between 2018 and 2021; (2) vitreoretinal surgery combined with silicon oil (1300 cst) implantation for complicated posterior segment disease; (3) at least 24-month follow-up period after MP-CPC; (4) no eye surgery was performed over a year before MP-CPC. Initially, we collected records of 13 patients, two of them were excluded. One patient was unable to attend follow-up visits after 9 months due to the COVID pandemic while the other patient had missed visits after 12 months due to pregnancy.

Before MP-CPC and in the follow-up period, all of the patients underwent routine ophthalmic examination: best corrected visual acuity (BCVA) was measured on Early Treatment Diabetic Retinopathy Study (ETDRS) chart after automatic refractometry, slit lamp examination, Goldmann applanation tonometry, funduscopy. Intra and postoperative complications were monitored. 

Indications for MP-CPC were: (1) for 9 eyes, intraocular pressure ≥21 mmHg despite using personally tolerable eyedrops and oral acetazolamide combination (i.e., at least 3 drugs in the eyedrops (beta blocker, carboanhydrase inhibitor, and alpha-adrenergic agonist) with or without oral carboanhydrase inhibitor or prostaglandine analog eyedrop); (2) concentrically narrowed visual field when intraocular pressure <21 mmHg for an eye of former premature patient, and this eye was managed with a combination of four drugs in the eyedrops. The reason for the visual field examination of the affected eye prior to MP-CPC was a suspected and confirmed concentric visual field narrowing due to retinopathy of prematurity and made even worse by secondary glaucoma. (3) The final indication was high intraocular pressure and severe pain in a blind eye of another former premature patient.

The treatment was performed with a Supra 810 nm diode laser (Quantel Medical) at 3 mm from the limbus with a G-probe device, skipping the 3 and 9 o’clock regions (energy: 2000 mW, duty cycle 31.3%, time: 2 × 80 s, sweeping motion: 10 s/hemisphere). After the micropulse laser treatment, all patients received steroid eyedrop therapy for 2 weeks in addition to the previously prescribed anti-glaucoma eyedrops. The patients were asked to come for control quarterly (every three months). All complications, whether intraoperative, early, or late postoperative, were recorded. Special attention was paid to whether the surgery did not result in visible laser burn or subconjunctival suffusion. Major postoperative complications were considered to be significant visual impairment associated with MP-CPC, hypotony, phthisis. Anterior segment inflammation and transient BCVA deterioration within the first 2 postoperative weeks due to inflammation were classified as mild complications. All participants were treated in accordance with the tenets of the Declaration of Helsinki. Written informed consent was obtained from all participants.

Statistical analysis was performed using IBM SPSS Statistics 25 (SPSS Inc., Chicago, IL, USA). Therapeutic answer was characterized by the eye pressure; number of medications at baseline; 3, 6, 9, 12, 15, 24, 36, and 48 months; and the number of MP-CPC sessions during the follow-up. The changes in the best corrected visual acuity were also analyzed. A significant visual deterioration was defined as 2 or more lines worsening in BCVA measured on ETDRS chart. Loss rate was calculated at each follow-up time point. Loss rate means the ratio of number of patients who attended a visit and number of all patients who could have been followed based on the starting date (2018–2021). Success was defined as the baseline eye pressure reduced at least 20%, and it should be ranged between 10 to 20 mmHg at the end of follow-up. If 2 measurements occurred in the given quarter, the last was included in the analysis. Success rate was calculated at each follow-up point (categorization: 0 for patients who met the criteria of success, 1 for patients with eyes that did not). Success rate is defined as number of eyes met the criteria of success at that given follow-up time point/total number of patients who could have been seen at that time. This means that (1) all patient who did not present were considered failures and, (2) due to the retrospective nature of the study, fewer patients were seen at the 36th and 48th month visits. The categorized values of eye pressure in the first 24 months were also estimated by chi-squared test. Wilcoxon signed rank test was performed to compare baseline and endpoint data of each eye. Friedman test was used to determine whether the repeated measurements of eye pressure and agent’s combination in the first 8 quarters differ from the baseline. Usage of oral acetazolamide at the end of follow-up of each patient was estimated by Fisher’s exact test. A *p*-value of < 0.05 was considered statistically significant. Datasets are deposited in Dryad database (DOI: https://doi.org/10.5061/dryad.v6wwpzh1b, accessed on 17 March 2023).

## 3. Results

Eleven eyes of eleven patients (eight males, three females) (Table 3) met the inclusion criteria. In all cases, the indication of MP-CPC was refractory glaucoma. At baseline, 7 patients had intraocular pressure more than 30 mmHg; one of them was blind. Three eyes were legally blind (BCVA ≤ 0.1) and another 3 eyes had 0.5 ≤ BCVA. Generally, the patients applied 3 agents (beta blocker, carboanhydrase inhibitor, and alpha-adrenergic agonist) in eyedrops, 3 patients added a prostaglandin analog, and 6 patients used an oral carboanhydrase inhibitor as well. Silicon oil was removed within 2 months to 2 years (median: 4.5 months); at the time of MP-CPC, a vitreous cavity in 4 eyes (36%) had been containing silicon oil for 1 to 10 years (median: 6.5 years). MP-CPC was performed from 8 months to 11 years after the silicon oil implantation (median: 3 years). All of the eyes underwent uncomplicated phacoemulsification and posterior chamber lens implantation either in the same session of vitrectomy (9 eyes) or before vitrectomy (2 eyes). Table 4 shows the loss and success rates at each follow-up time point, respectively.

*Comorbidities of secondary glaucoma*. The vitrectomies were performed between 2008 and 2019; the indications for silicon oil implantation were the following: retinal detachment with proliferative vitreoretinopathy in 5 eyes. Out of them one patient manifested rheumatoid arthritis (RA) and consecutive uveitis in both eyes. Another patient has ocular hypertension in the fellow eye. Two former premature patients suffered from retinal detachment caused by circular remnant of retinopathy of prematurity (ROP): one of them was blind for the fellow eye, the other patient’s operated eye had perforating sclera injury previously. In another patient, 2 quadrants large retinal tear developed with retinal detachment 8 months after an uncomplicated phacoemulsification for congenital cataract. The next patient had a perforating cornea injury and underwent vitrectomy combined perforating keratoplasty using a temporary prosthesis. Two type 1 insulin dependent diabetic patients had severe proliferative diabetic retinopathy.

*Complications*. Mild acute complications, such as hyperemia or mild pain, occurred in seven and one patients, respectively. No severe complications were observed either intraoperatively or postoperatively. In the acute postoperative period, mild iritis was detected that resolved within 10 days using a steroid eyedrop. None of the patients developed a phthisis or hypotony during follow-up. No eyes became blind.

*Visual acuity*. Except for 2 eyes, the original visual acuity could be preserved. One line worsening in BCVA occurred in 2 eyes, one line improvement was detected in another 2 eyes, and BCVA did not change in 4 eyes. In the remaining one eye was blind at the time of MP-CPC. Significant BCVA loss occurred in 2 eyes. One of them underwent an unsuccessful pars plana vitrectomy for retinal detachment 10 years before MP-CPC. The five lines of the deterioration of BCVA is determined by not only proliferative vitreoretinopathy, but also uveitic episodes due to rheumatoid arthritis. Rheumatoid arthritis was diagnosed a year after MP-CPC. In the first 2 years of the 5-year follow-up period of MP-CPC, iridocyclitis with macular edema developed 3 times in both eyes. The patient was unable to attend control examinations due to the COVID-19 pandemic. Three lines worsening in BCVA also occurred in the fellow eye, and secondary glaucoma was detected and required a shunt implantation. The other patient’s eye underwent a perforating keratoplasty combined pars plana vitrectomy for retinal detachment after a perforating cornea injury. In this case, the 5 lines of impairment in BCVA is explained by the corneal haze of the donor tissue.

**Figure 1:**
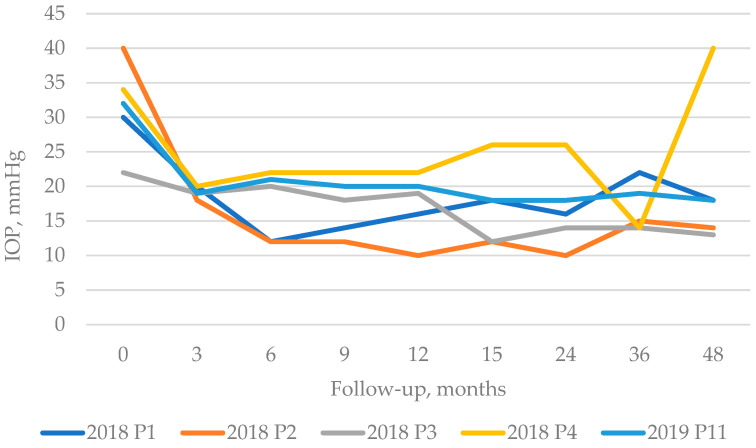
Values of eye pressure of 5 representative patients who could have been followed for at least 48 months, at each follow-up time point. (The color explanatory lines demonstrate the date of MP-CPC and the patient’s identity number.)

**Figure 2:**
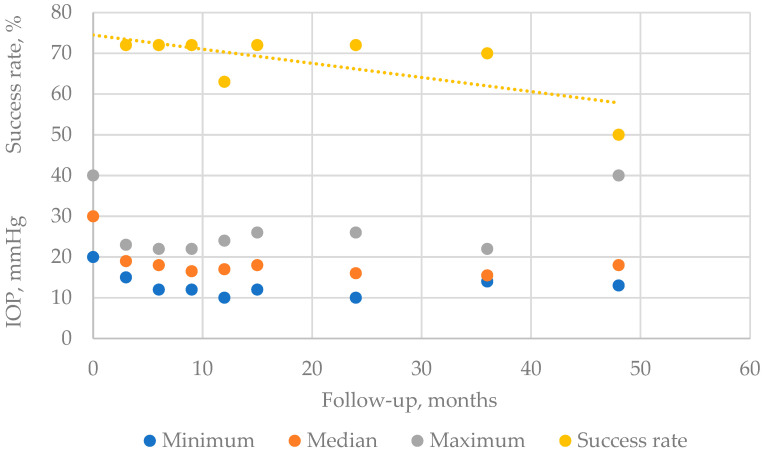
Changes in the intraocular pressure (IOP) at months 24, 36, 48: 11, 8, and 5 eyes, respectively. Success rate (including the second session of MP-CP; median: 72%, 50–72) does not differ significantly through the first 2-year follow-up, *p* = 0.99. Three eyes required second session of MP-CPC.

*Intraocular pressure.* In the first 2 years, the intraocular tension could be measured regularly: in a 3-month period in the first 15 months, after said period, and in a 6–8 month period, respectively. Figure 1 shows the evolution of eye pressure during the follow-up period. Table 4 summarizes the loss rates at each follow-up time point. Seven patients could be followed for more than 3 years (40–52 months). The median values of eye pressure were all below 20 mmHg (Figure 2). Except one eye, the others achieved the range of 20 mmHg or less with an approximately 20% reduction at the end of the follow-up period for each eye (Wilcoxon test, *p* = 0.004). Three patients required second session of MP-CPC, 6, 8, and 14 months after the first treatment, respectively (re-treatment rate 27%). The success rate of MP-CPC is 73% at the end of the first 2 years (based on the eye pressures collected in the first 24 months; Friedman test *p*-value is 0.013). Two patients achieved a 20% reduction; however, they even had an IOP above 20 mmHg (both of them had 26 mmHg with 23.50% IOP reduction). The third patient’s eye pressure was under 20 mmHg, but it was only a 10% reduction (18 mmHg). Out of these 3 patients, one eye failed at the end of 48 months of follow-up. This ROP eye was blind and contained silicon oil for 10 years at the time of MP-CPC. *The combination of agents* in eyedrops could be reduced in one eye, and it was achieved only in the first 3 months. For *oral acetazolamide*, at baseline, 6 patients used oral acetazolamide while at the end of follow-up of each patient, only 1 patient applied it (Table 3). The result is not significant (*p* = 0.0635), and the odds ratio is 12.00 at CI 95 1.117 to 128.9. We have to mention that at the 24-month follow-up point, no patient required oral acetazolamide. One patient required oral acetazolamide medication permanently over a year after MP-CPCP.

## 4. Discussion

Traditionally, cyclodestructive procedures are applied for otherwise non-treatable glaucoma. Transscleral CPC has a significant IOP lowering effect at 1, 5, and even 10 years. However, 51.5% of the treatments failed by the end of 10 years, and most failures occurred within the first year (40%). A high complication rate is reported in connection with this type of cyclodestructive procedure, such as a visual loss of two lines or more in 60% of patients and hypotony in 4% of eyes [8]. A subcyclo treatment uses a non-destructive laser device avoiding the side effects of the traditional transscleral procedure allowing a more precise management of the thermal effect on the targeted tissues [9].

In this retrospective preliminary study, we aimed the evaluation of IOP control using subcyclo treatment in eyes with refractory secondary open angle glaucoma caused by silicon oil implantation after vitrectomy surgery. The cases had in common that they all turned out to be complicated (e.g., complicated retinal detachment, repeated vitrectomies, former ROP patient, perforating eye injury in history, proliferative diabetic retinopathy and rheumatoid arthritis associated uveitis), and all patients underwent silicon oil implantation. Despite the fact that the silicon oil was removed, filtration surgery to reduce IOP (e.g., trabeculectomy) was not an option in these cases due to the residual emulsified silicon oil particles. Our basic aim with the MP-CPC treatment was to reduce the patients’ pain by reducing the intraocular pressure and to preserve residual visual acuity by avoiding potential complications (e.g., phthisis, hypotony).

*IOP lowering.* Our results confirm a significant IOP lowering effect of this subthreshold method, preserving visual performance safely even in eyes with previous vitrectomy surgery with silicone oil implantation (Table A1 in Appendix A). The reduction in IOP was found to be significant. Regarding *medications*, contrary to the literature data (see Table A1), the change in the number of anti-glaucoma agents in the eyedrops administered was not significant compared to the baseline values. In these complicated cases, we found it very difficult to change the existing therapy because the delicate balance of previously well-adjusted IOP values can easily be upset. The pathogenesis of secondary glaucoma is complex, and in addition to the emulsified silicone oil, several other factors are involved (e.g., autoimmune uveitis, prematurity, proliferative diabetic retinopathy, ocular trauma). We had a patient who was on continuous oral acetazolamide therapy for over one year. The odds ratio in Table 3 shows that after MP-CPC, there is a twelve-fold increase in the likelihood of getting rid of oral acetazolamide. In these cases, it was not possible to achieve a result with MP-CPC that would have led to a significant reduction or elimination of the number of eye drops used. At the same time, however, it also seems that MP-CPC could be an effective adjuvant therapy in such cases. For *visual acuity*, in the majority of the eyes, the change in BCVA values was not significant at the end of follow-up time; two or more lines worsening in BCVA were not detected. In two eyes, the severe visual impairment is definitely not associated with MP-CPC, and it could be explained by a difficult clinical picture. As the visual acuity in these complex cases is affected by many factors, we have not calculated a success rate for BCVA, and the change in BCVA values was not significant at the end of follow-up time. Regarding *complications*, we detected only mild acute complications, such as hyperemia or mild pain. No IOP spike, fibrinous reaction, or iritis was observed. We found no severe complications, such as prolonged hypotony, phthisis, cystic macular edema, or keratopathy. Severe visual deterioration in two patients can be explained by ocular pathology other than secondary glaucoma induced optic neuropathy (i.e., opacity of the donor cornea, autoimmune uveitis, and proliferative vitreoretinopathy). We cannot comment on a possible increased cataractogenesis since all of the patients underwent lens surgery several years before MP-CPC. Based on the literature data, possible complications of MP-CPC were collected in Table 2, and they occurred within the following frequency ranges regardless of the etiology (see details in Table A1): (1) severe complications: significant BCVA deterioration (4.5–44%), prolonged hypotony (1.1–13%), phthisis (2.5–5%), cystic macular edema (1.4–5%), keratopathy (1.6–18%), hyphaema (17,5%), and vitreous haemorrhage (0.3%); (2) mild complications: acute hyperemia (73%), mild pain (30%), IOP spike (10%), fibrinous reaction (0.7–3%), and iritis (1.8%). In the light of the literature data, it can be concluded that the symptoms considered as mild complications were within the known prevalence values in our study (hyperemia in 63.6% and mild pain in 9.1%).

*The literature data on secondary glaucoma developed after the vitreoretinal procedure.*Table A1 provides details of MP-CPC studies on secondary glaucoma previously published in the literature [10,11,12,13,14,15,16,17,18,19,20,21,22,23,24,25,26,27,28,29,30,31,32,33,34,35]. The literature data are difficult to compare because the definition of success varies from study to study, the etiology is diverse, and therefore the success rates calculated at the end of follow-up period show a wide variation (from 11.0% to 95.7%; for details see Table A1). Most of the studies were retrospective; 6 studies were planned prospectively with the prospective studies being sporadic. We selected for inclusion in the discussion those publications in which we found references to MP-CPC for the treatment of secondary glaucoma, especially which developed after vitreoretinal procedure. Regarding the *prospective studies,* except for the study of Zbiba et al., 2022 [10], the others (Tan et al., 2010 [35], Aquino et al., 2015 [34], Barac et al., 2018 [26], and Jammal et al., 2019 [19]) were not addressed to analyze silicon oil-induced glaucoma as a different subgroup. In these studies, the success rates ranged from 52–72%; in one study the success rate was not applicable. Zbiba et al. [10] investigated only eyes with refractory stage silicon oil-induced glaucoma. Out of 33 eyes, 10 eyes contained silicon oil at the time of MP-CPC, and the follow-up was 12 months. The lowest eye pressure (16.5 mmHg) was measured at the end of the first week. After, it ranged between 19.27–19.97 mmHg. The success rate was 93.93% with a percentage of eye pressure lowering 45%.

As for the results of the *retrospective studies*, 2 studies were found that included vitrectomized eyes. Lim et al., 2021 [13] investigated the outcomes of MP-CPC for secondary glaucoma. Of the 43 eyes treated, 3 were vitrectomized. At the end of the mean follow-up of 28.9 months, the success rate was 39.5%. Tekeli et al., 2021 [14] compared the outcomes of primary open angle glaucoma, pseudoexfoliation, and secondary glaucoma groups, respectively. The success rates were the lowest in the secondary glaucoma group at all IOP criteria (A, B, and C), and the retreatment rate was the highest (41.2%) in this group. The data in the literature suggest that silicone oil-induced secondary glaucoma is one of the groups with the worst prognosis. As it was mentioned above, a number of other factors can make a patient’s condition worse; therefore, each patient requires personalized treatment.

It is difficult to compare the results of our study with the aforementioned studies, as most of them did not include vitrectomized eyes as a separate subgroup, and where it was analyzed as a separated subgroup, it was mentioned together with several other types of secondary glaucoma. However, our success rate was lower compared to the results in Zbiba et al.’s study. It was stable through the follow-up period. The differences might arise from the long-term history of silicon oil usage and the complex pathogenesis of glaucoma, including among other autoimmune uveitis, prematurity, trauma, and severe diabetic proliferative retinopathy. 

*The limitations of our study* are the retrospective design and the small number of patients. However, all of the patients underwent vitrectomy combined with silicone oil implantation, and the presented case series may not be representative for secondary glaucoma-induced silicone oil since many other factors are also implicated in the development of secondary glaucoma. This report should be considered as a preliminary study that requires further data validated from a greater number of patients treated in different centers.

*In conclusion*, the manuscript provides real-life data on an uncommon but difficult-to-treat type of secondary glaucoma that develops after vitreoretinal surgery, related to silicone oil implantation but with a pathogenesis that is more complex than only silicone oil-induced. We found MP-CPC to be a safe and effective adjuvant treatment modality even in difficult cases, such as vitrectomized eyes.

## Figures and Tables

**Table 1:** Indications for MP-CPC based on previously published studies (for details see Table A1).

1. Childhood glaucomas o Primary congenital glaucoma o Juvenile glaucoma
2. Primary open angle glaucoma (POAG)
3. Secondary glaucomas A. Secondary open angle glaucomas caused by ocular disease o Exfoliative (pseudoexfoliative) glaucoma o Pigmentary glaucoma o Uveitic glaucoma o Neovascular glaucoma B. Secondary open angle glaucoma due to ocular trauma C. Iatrogenic secondary open angle glaucomas o Corticosteroid treatment o Ocular surgery after cataract surgery after perforating keratoplasty (PKP) after pars plana vitrectomy (PPV) with silicon oil implantation
4. Primary angle closure glaucoma Chronic angle closure glaucoma (CACG)

**Table 2:** Complications reported in previously published studies following MP-CPC treatment (for details see Table A1).

o hyperemia o pain o chemosis o BCVA deterioration of ≥ 2 lines o intraocular pressure spike o prolonged hypotony o phthisis bulbi o cystic macular edema	o fibrinous/uveitic reaction o hyphaema o keratopathy o corneal edema o cataract o iritis o tonic pupil o vitreous haemorrhage

**Table 3:** Demographic data, results of Wilcoxon signed rank test and Fisher’s exact test.

Parameter	When	Median (Min–Max)	*p* Value
Age (years) Follow-up (months)	at the first session of MP-CPC	55 (21–67)	-
		46 (24–52)	-
Best corrected visual acuity	at baseline	0.3 (0–1.0)	0.462
	at the end of follow-up	0.3 (0–1.0)	
Intraocular pressure (mmHg)	at baseline	30 (20–40)	0.004
	at the end of follow-up	16 (13–40)	
No. of agents in the eyedrops	at baseline	3 (3–4)	0.655
	at the end of follow-up	3 (2–4)	
Oral acetazolamide (No. of patients)	at baseline	6	0.063 (odds 12.0; 95% CI: 1.117 to 128.9)
	at the end of follow-up	1	

**Table 4:** Loss and success rates at each follow-up time point, respectively. Calculation of loss rate: patient who attended a visit/all patients followed, depending on the date of first session of MP-CPC (2018–2021). A different patient was always missing at each visit. Calculation of success rate: number of eyes met the criteria of success/number of patients who could have been seen at follow-up time point. All patients who did not visit were considered failures.

Visit date (months)	3	6	9	12	15	24	36	48
Loss rate, % (calculation)	0 (0/11)	9 (1/11)	9 (1/11)	9 (1/11)	0 (0/11)	0 (0/11)	20 (2/10)	37 (3/8)
Success rate, % (calculation)	72 (8/11)	72 (8/11)	72 (8/11)	63 (7/11)	72 (8/11)	72 (8/11)	70 (7/10)	50 (4/8)

## Data Availability

The data presented in this study are openly available in Dryad database at DOI: https://doi.org/10.5061/dryad.v6wwpzh1b, accessed on 17 March 2023).

## Appendix A

**Table A1:** Summary of previous MP-CPC studies.

Author, Year Study Type	Study Details	Success Parameters (D: Day, W: Week, M: Month, Y: Year)	Results
Zbiba et al. [10], 2022 Prospective	**Number of eyes (number of patients):** 33 (33) **Etiology (number of eyes):** Silicon oil-induced glaucoma (33) **Follow-up (months):** 12 **Preop. IOP (mmHg):** 37.94	**Definition of success (IOP, lowering):** IOP↓ > 20% or IOP < 21 mmHg with a decrease of medications without visual acuity decline at the final follow-up **Success rate (%):** M6: 93.93 M12: remained stable	*Achieved IOP ± SD (mmHg):* D1: 19.03 ± 10.98 D7: 16.5 ± 6.17 D15: 19.45 ± 9.73 M1: 19.27 ± 8.33 M3: 19.39 ± 9.52 M6: 19.97 ± 10.03 M12: 19.70 ± 9.58 •Number of anti-glaucoma medications decreased from 3.88 to 3.15 at M12. •Complications: moderate pain (10) hyperemia (24) chemosis (2) severe anterior chamber inflammation (1)
Chamard et al. [11], 2021 Retrospective	**Number of eyes (number of patients):** 94 (94) **Etiology (number of eyes):** POAG (84) Steroid-induced glaucoma (3) Inflammatory glaucoma (3) Traumatic glaucoma (2) Pigmentary glaucoma (1) Glaucoma after iris-clipped IOL implantation (1) **Follow-up (months):** 6 **Preop. IOP (mmHg):** 24.9	**Definition of success (IOP, lowering):** IOP 5–21 mmHg and preop. IOP↓ ≥ 20% (without any retreatment and visual acuity better than negative light perception) **Success rate (%):** W1: 64.4 M1: 57.5 M3: 45.3 M6: 45.5	*Achieved IOP ± SD (mmHg):* M6: 18.9 ± 6.3 •15 patients (16%) underwent early (≤6 months) retreatment at a median postoperative delay of 4.4 ± 1.3 months. •19 patients (20%) underwent late (≥6 months) retreatment at a median postoperative delay of 10.2 ± 3.6 months.
de Crom et al. [12], 2021 Prospective	**Number of eyes (number of patients):** 141 (136) **Etiology (number of eyes):** POAG (99) Secondary glaucoma (42) Neovascular (13) Postvitrectomy (10) Uveitis (7) Trauma (6) Complicated phaco procedure (3) Secondary IOL implant (2) Perforating keratoplasty (1) **Follow-up (months):** 24 **Preop. IOP (mmHg):** 23.5	**Definition of success (IOP, lowering):** IOP↓ > 20% or a decrease in the number of IOP-lowering medications with stable target IOP **Success rate (%):** M12: 72.3 M18: 82.2 M24: 80.0	*Achieved IOP ± SD (mmHg):* M12: 16.8 ± 8.4 M18: 17.0 ± 7.8 M24: 16.8 ± 9.2 •Number of anti-glaucoma medications decreased from 3.0 ± 1.7 to 2.2 ± 1.5 at M24. •Complications: cystic macular edema (2) hypotony maculopathy (1) fibrinous/uveitic reaction (1) rejection of corneal graft (1)
Lim et al. [13], 2021 Retrospective	**Number of eyes (number of patients):** 43 (43) **Etiology (number of eyes):** NVG (18) POAG (9) Uveitic glaucoma (3) Silicon oil glaucoma (3) PACG/CACG (2) Postkeratoplasty glaucoma (1) Postvitrectomy glaucoma (1) Steroid-included glaucoma (1) Pediatric/juvenile glaucoma (2) Aphakic glaucoma (1) Phacomorphic glaucoma (2) **Follow-up (months):** 28.9 **Preop. IOP (mmHg):** 35.2	**Definition of success (IOP, lowering):** IOP: 6–21 mmHg or preop. IOP↓ ≥ 20% without an increase in glaucoma medications from baseline without further glaucoma reoperation ≤3 total MPTCP episodes **Success rate (%):** Y1: 36.4 Y2: 42.9 Y3: 32.0 latest: 39.5	*Achieved IOP ± SD (mmHg):* Y1: 27.8 ± 13.7 *(p = 0.004)* Y2: 27.4 ± 12.4 *(p = 0.003)* Y3: 31.8 ± 13.2 *(p = 0.35)* latest: 27.1 ± 13.8 *(p = 0.002)* •Number of preop. anti-glaucoma medications: 3.3 ± 0.9 at baseline and 2.8 ± 1.3 at the final follow-up *(p = 0.007)*. •Complications: prolonged hypotony (3) phthisis bulbi (2) BCVA deterioration of ≥2 lines (14) •The median survival time of repeated MPTCP was 4.6 months.
Tekeli et al. [14], 2021 Retrospective	**Number of eyes (number of patients):** 96 (96) **Etiology (number of eyes):** group 1: POAG (32) group 2: Pseudoexfoliation glaucoma (30) group 3: Secondary glaucoma (34): Post-PKP glaucoma (14) Post-PPV glaucoma (9) Neovascular glaucoma (6) Pseudophakic glaucoma (3) Uveitis/Inflammation-associated gl. (2) **Follow-up (months ± SD):** 14.2 ± 3.9 **Preop. IOP (mmHg):** group 1: 34.3 group 2: 34.2 group 3: 35.7	**Definition of success (IOP, lowering):** Criteria A: IOP ≤ 18 mmHg and preop. IOP↓ ≥ 20% Criteria B: IOP≤15 mmHg and preop. IOP↓ ≥ 25% Criteria C: IOP ≤ 12 mmHg and preop. IOP↓ ≥ 30% (with/without anti-glaucoma medications) **Success rate (%):** Criteria A: 82.4 Criteria B: 73.6 Criteria C: 61.4 group 1: 68.75 group 2: 66.6 group 3: 64.7	*Achieved IOP ± SD (mmHg):* group 1: M1: 22.50 ± 8.85 M3: 20.38 ± 8.05 M6: 21.22 ± 7.46 M9: 20.65 ± 7.21 M12: 19.72 ± 6.71 group 2: M1: 23.29 ± 10.63 M3: 22.12 ± 6.56 M6: 20.84 ± 7.79 M9: 21.25 ± 8.16 M12: 21.50 ± 7.57 group 3: M1: 26.50 ± 11.02 M3: 20.26 ± 6.29 M6: 20.20 ± 9.17 M9: 20.74 ± 6.71 M12: 21.88 ± 7.65
Kaba et al. [15], 2020 Retrospective	**Number of eyes (number of patients):** 342 (214) **Etiology (number of eyes):** POAG (223) CACG (43) Neovascular glaucoma (36) NTG (26) OHT (22) **Follow-up (months):** 12 **Preop. IOP (mmHg):** 19.8	**Definition of success (IOP, lowering):** preop. IOP↓ ≥ 20% **Success rate (%):** 67.8	*Achieved IOP ± SD (mmHg) and preop. IOP↓ (%):* M1: 15.3 ± 6.0; 22.7% M3: 15.8 ± 6.6; 20.2% M6: 15.7 ± 5.7; 20.7% M12: 15.1 ± 6.3; 23.7% (*p < 0.0001* for all time points) •One or more repeated MPCPC treatment was administered of 14.3% of the cohort. •Number of preop. anti-glaucoma medications: 1.6 ± 1.1 at baseline and 1.6 ± 1.1 at M12 *(p = 0.91)*. •Adverse events at M1: visual acuity loss (61) IOP spike (34) cataract (7) iritis (6) symptomatic mydriasis (6) hypotony (4) vitreous hemorrhage (1)
Preda et al. [16], 2020 Prospective	**Number of eyes (number of patients):** 100 (97) **Etiology (number of eyes):** POAG Pseudoexfoliation glaucoma Neovascular glaucoma **Follow-up (months):** 18 **Preop. IOP (mmHg):** 39.14	**Definition of success (IOP, lowering):** IOP: 6–21 mmHg and preop. IOP↓ ≥ 30% **Success rate (%):** M18: 90.91 (IOP < 26 mmHg) 70.00 (IOP between 26–30 mmHg) 65.63 (IOP between 31–49 mmHg) 84.62 (IOP > 50 mmHg)	*Achieved IOP ± SD (mmHg):* W1: 22.77 ± 10.48 (41.82% reduction; *p < 0.001*) M1: 23.81 ± 9.44 M3: 24.27 ± 9.17 M6: 23.09 ± 8.47 M12: 22.76 ± 8.14 M18: 22.77 ± 8.13 (*p < 0.001* at all time points) •Number of preop. anti-glaucoma medications decreased from 2.63 ± 0.87 to 1.78 ± 0.95 at M18 (decrease of 32%). •No complications
Al Habash et al. [17], 2019 Prospective	**Number of eyes (number of patients):** 71 (69) **Etiology (number of eyes):** Neovascular glaucoma (24) POAG (15) Secondary glaucoma (14) Keratoplasty (6) Aphakia (3) Keratoprosthesis (1) Cyst excision (1) ICE syndrome (1) Trauma (1) Unknown etiology (1) CACG (9) Pseudoexfoliation glaucoma (4) Microphthalmos (2) Uveitic glaucoma (2) Congenital glaucoma (1) **Follow-up (months):** 24 **Preop. IOP (mmHg):** 35.0	**Definition of success (IOP, lowering):** IOP: 6–21 mmHg or preop. IOP↓ ≥ 30% without vision loss of “light perception”, with no secondary glaucoma intervention, and without an increase in the number of medications **Success rate (%):** W2: 90.0 M3: 91.4 M6: 95.7 M9 and 12: remained stable	*Achieved median IOP (mmHg):* W2: 10 M1: 13 M3: 16 M6: 16 M9: 16 M12/last follow-up: 16 •Number of preop. anti-glaucoma medications decreased from 5 to 4 at M12/last follow-up *(p < 0.001)*. •2 eyes received retreatment, 3 eyes received other surgical interventions. •Complications: inflammation (1) tonic pupil (4)
Garcia et al. [18], 2019 Retrospective	**Number of eyes (number of patients):** 116 **Etiology (number of eyes):** POAG (66) CACG (7) Congenital (6) Juvenile (5) Pseudoexfoliative glaucoma (6) Other (5) Low tension glaucoma (8) Neovascular glaucoma (3) Post-traumatic glaucoma (3) Mixed mechanism glaucoma (2) Pigment dispersion glaucoma (2) Uveitis/Inflammation-associated gl. (2) Aphakia (1) **Follow-up (months ± SD):** 6.3 ± 3.4 **Preop. IOP (mmHg):** 22.2	**Definition of success (IOP, lowering):** IOP: 6–21 mmHg or preop. IOP↓ ≥ 30% for any 2 consecutive visits after 3 postoperative months no subsequent glaucoma surgery, and no loss of light perception vision or vision-threatening severe complications **Success rate (%):** M3: 93.1 M6: 74.3 M9: 67.5 M12: 59.6	*Achieved IOP ± SD (mmHg):* D1: 15.3 ± 6.9 M1: 16.0 ± 6.6 M3: 15.8 ± 6.9 M6: 16.1 ± 7.0 M9: 14.9 ± 5.3 M12: 17.0 ± 4.2 •Mean IOP was significantly lower at all postop. visits and the final follow-up (15.3 ± 6.6 mmHg) compared with preop. IOP *(p < 0.01)*. •Number of anti-glaucoma medications decreased from 3.2 to 2.5 at final follow-up *(p < 0.01)*.
Jammal et al. [19], 2019 Prospective	**Number of eyes (number of patients):** 21 (21) **Etiology (number of eyes):** Neovascular glaucoma (11) POAG (7) Silicon oil induced glaucoma (2) Post-traumatic glaucoma (1) **Follow-up (months):** 12 **Preop. IOP (mmHg):** 33.38	**Definition of success (IOP, lowering):** IOP: 6–21 mmHg or preop. IOP ↓ ≥ 30% (with/without anti-glaucoma medications) **Success rate (%):** D1: 71.4 M1: 76.2 M3: 57.1 M6 55.6 M12: 66.7	*Achieved IOP ± SD (mmHg) and preop. IOP ↓ (%):* W1: 18.5; 45% M1: 17.6; 38% M3: 19.0; 34% M6: 22.3; 34% M12: 18.9; 42% •Number of anti-glaucoma medications decreased from 3.5 to 2.0 at M12 *(p < 0.044)*. •7 patients (33.3%) needed repeated laser treatment: 3 patients: CW-TSCPC 1 patient: MP-TSCPC 3 patients: not participated
Nguyen et al. [20], 2019 Retrospective	**Number of eyes (number of patients):** 95 (95) **Etiology (number of eyes):** POAG (51) Pseudoexfoliative glaucoma (24) CACG (15) Congenital or juvenile glaucoma (5) **Follow-up (months):** 12 **Preop. IOP (mmHg):** 25.1	**Definition of success (IOP, lowering):** preop. IOP↓ ≥ 20% **Success rate (%):** 76.8	*Achieved IOP ± SD (mmHg):* W1: 15.1 ± 7.4 (*p = 0.002)* M1: 14.1 ± 5.6 *(p = 0.001)* M3: 16.2 ± 4.5 *(p = 0.003)* M6: 16.1 ± 4.4 *(p = 0.001)* M12: 17.5 ± 5.1 *(p = 0.004)* •Preop. IOP decreased 30.3% to M12. •Number of anti-glaucoma medications decreased from 3.0 ± 1.1 to 1.4 ± 1.0 at M12. •Complications: mild hypotony (10) keratopathy (10) long-term hypotony (1)
Souissi et al. [21], 2019 Retrospective	**Number of eyes (number of patients):** 37 (37) **Etiology (number of eyes):** POAG (17) Uveitis-associated glaucoma (3) Congenital glaucoma (3) Post-traumatic glaucoma (3) Pigmentary glaucoma (3) Pseudoexfoliative glaucoma (2) Neovascular glaucoma (2) Juvenile glaucoma (1) Malignant glaucoma (1) CACG (1) Sturge-Weber-Krabbe syndrome (1) **Follow-up (months ± SD):** 9.7 ± 3.9 **Preop. IOP (mmHg):** 28.7	**Definition of success (IOP, lowering):** Criterion A: IOP: 6–18 mmHg or preop. IOP↓ ≥ 20% Criterion B: IOP: 6–15 mmHg or preop. IOP↓ ≥ 25% Criterion C: IOP: 6–12 mmHg or preop. IOP↓ ≥ 30% no need to add anti-glaucoma drop to the therapy or visual acuity is not reduced due to complications or fluctuating intraocular pressure or no need for glaucoma surgery (except MP-TSCPC) **Success rate (%):** Criterion A: M3: 76; M6: 46; M9: 35 Criterion B: M3: 43; M6: 32; M9: 27 Criterion C: M3: 30; M6: 16; M9: 11	*Achieved IOP ± SD (mmHg) and preop. IOP↓ (%):* W1: 16.2 ± 8.4; 44% M1: 21.0 ± 8.4; 27% M3: 18.5 ± 8.7; 36% M6: 18.4 ± 6.8; 36% M12: 18.5 ± 7.0; 36% total IOP decrease: 43.7% •Preop. IOP decreased 18.5 (±7.0) mmHg (36%) at M12 *(p < 0.01)*. •Number of preop. anti-glaucoma medications decreased from 4.7 ± 1.9 to 3.6 ± 1.9 at M12 *(p < 0.05)*. •5 patients (13.5%) needed repeated MP-TSCPC •12 patients (32.4%) non-responder •6 patients: filtration surgery •2 patients: Ahmed shunt •2 patients: CW-TSCPC
Subramaniam et al. [22], 2019 Retrospective	**Number of eyes (number of patients):** 61 (57) **Etiology (number of eyes):** Post-PKP glaucoma (57) **Follow-up (months ± SD):** 12 **Preop. IOP (mmHg):** 28	**Definition of success (IOP, lowering):** n/a **Success rate (%):** n/a	*Achieved IOP ± SD (mmHg) and* *preop. IOP↓ (%):* M1: 17 ± 7; 30% M3: 17 ± 8; 30% M6: 18 ± 9; 31% M12: 15 ± 5;34% •Complications: enucleation (1) keratopathy (1) hypotony (1) •Glaucoma filtration surgery (6)
Varikuti et al. [23], 2019 Retrospective	**Number of eyes (number of patients):** 61 (46) **Etiology (number of eyes):** POAG (51) CACG (5) Other (4) **Follow-up (months ± SD):** 10.2 ± 3.1 **Preop. IOP (mmHg):** 25.69	**Definition of success (IOP, lowering):** IOP: 6–21 mmHg or preop. IOP↓ ≥ 20% or BCVA decrease ≤ 2 lines or no need for glaucoma surgery (except MP-TSCPC) **Success rate (%):** M1: 74.1 M3: 83.6 M6: 84.2 M12: 75.0	*Achieved IOP ± SD (mmHg) and* *preop. IOP↓ (%):* M1: 16.69 ± 4.79; 75.86% M3: 15.20 ± 4.15; 90.16% M6: 15.33 ± 3.46; 86.21% M12: 15.45 ± 3.74; 85.42% •Preop. IOP↓ was 40.2% at M12. •≥20% preop. IOP↓: 85.4% of patients •Therapy decreased≥1 anti-glaucoma drug 79.6% of patients •Decrease in BCVA was not significant *(p < 0.05).*
Zaarour et al. [24], 2019 Prospective	**Number of eyes (number of patients):** 75 (69) **Etiology (number of eyes):** POAG (26) Secondary glaucoma (10) Post-PKP glaucoma (7) CACG (6) Congenital glaucoma (5) Unknown origin (5) Neovascular glaucoma (4) Mixed mechanism glaucoma (4) Pseudoexfoliative glaucoma (3) Aphakia (2) Aniridia (1) Fuchs heterochromic iridocyclitis (1) Juvenile glaucoma (1) **Follow-up (months ± SD):** 13.2 ± 3.0 **Preop. IOP (mmHg):** 26	**Definition of success (IOP, lowering):** IOP: 6–21 mmHg or preop. IOP↓ ≥ 20% (with/without anti-glaucoma medications) **Success rate (%):** D1: 65.3 W1: 92.0 M1: 76.0 M3: 80.6 M6: 81.4 M9: 78.5 M12: 73.3 M15: 66.0	*Achieved IOP ± SD (mmHg) and* *preop. IOP↓ (%):* D1: 21.0 ± 6.9; 16% W1: 13.8 ± 5.6; 44% M1: 18.0 ± 7.7; 26% M3: 18.4 ± 7.1; 24% M6:16.7 ± 6.2; 29% M9: 15.1 ± 4.1; 37% M12 15.7 ± 5.3; 32% M15: 14.8 ± 5.5; 35% •Number of anti-glaucoma medications decreased from 0.72 to 0.15 at M12 *(p < 0.008)*. •No complications
Abdelrahman et al. [25], 2018 Prospective	**Number of eyes (number of patients):** 45 (36) MP-CPC: 17 (13) CW-CPC: 28 (23) **Etiology (number of eyes):** Congenital glaucoma (11/15) Aphakia/Pseudophakia (3/9) Aniridia (2/1) Peter’s anomaly (1/0) Microspherophakia (0/2) Sturge-Weber syndrome (0/1) **Follow-up (months ± SD):** 6 **Preop. IOP (mmHg):** MP-CPC: 28.3 CW-CPC: 27.5	**Definition of success (IOP, lowering):** Complete success: IOP: 5–21 mmHg with no glaucoma progression Qualified success: IOP < 21 mmHg and preop. IOP↓ ≥ 20% and/or reduction in the number of medications Failure: above mentioned criteria were not achieved, or subsequent CPC or other procedure was needed, or complication occurred **Success rate (%):** MP-CPC/CW-CPC: W2: 12.1/15.9 M2: 16.8/17.7 M3: 15.5/19.4 M6: 16.4/17.9	*IOP reduction (%):* MP-CPC: 63% ± 28% CW-CPC: 67% ± 25% *Success rates at M6 (eyes) (complete/qualified/failure):* MP-CPC: 1/12/5 eyes CW-CPC: 5/13/14 eyes Difference in failure rates between both groups was not significant. •Complications: MP-CPC: 1 eye: hypotony (at 2 weeks) CW-CPC: 3 eyes: hypotony (1 eye phthisis)
Barac et al. [26], 2018 Prospective	**Number of eyes (number of patients):** 22 **Etiology (number of eyes):** Silicon oil associated (8) POAG (5) Neovascular glaucoma (5) PACG (2) Juvenile glaucoma (1) Post-traumatic glaucoma (1) **Follow-up (months ± SD):** 6 **Preop. IOP (mmHg):** 35.23	**Definition of success (IOP, lowering):** n/a **Success rate (%):** n/a	*Achieved IOP (mmHg) and* *preop. IOP↓ (%):* W1: 17.73; 49.67% M1: 21.81; 38.09% M3: 22.34; 36.58% M6: 23.56; 33.12% •Number of anti-glaucoma medications decreased from 3.14 to 3.10 at M6. •Number of acetazolamide doses per day decreased from 1.18 to 0.27 at M6. •4 patients needed repeated laser treatments. •Complications: blurriness (5) keratopathy (4) decrease of BCVA (1)
Lutic et al. [27], 2018 Prospective	**Number of eyes (number of patients):** 50 (32) **Etiology (number of eyes):** POAG (40) Neovascular glaucoma (3) Uveitis-associated glaucoma (2) Juvenile glaucoma (2) Pigmentary glaucoma (2) Post-traumatic glaucoma (1) **Follow-up (months ± SD):** 12 **Preop. IOP (mmHg):** 26.27	**Definition of success (IOP, lowering):** n/a **Success rate (%):** n/a	*Achieved IOP ± SD (mmHg):* D1: 15.9 ± 5.72 W1: 13.72 ± 4.31 W6: 15.81 ± 3.69 M3: 15.94 ± 5.66 M6: 16.32 ± 5.24 *Preop. IOP↓ (%):* 37% at M12. •Number of anti-glaucoma medications decreased from 3.14 to 2.56 at M12. •9 eyes required second laser treatment •No complications
Sanchez et al. [28], 2018 n/a	**Number of eyes (number of patients):** 22 (17) **Etiology (number of eyes):** Congenital glaucoma (7) Pseudoexfoliative glaucoma (5) Aphakia (3) Post-PKP glaucoma (2) POAG (2) Mixed mechanism glaucoma (2) Juvenile glaucoma (1) **Follow-up (months ± SD):** 7.9 **Preop. IOP (mmHg):** 26.3	**Definition of success (IOP, lowering):** IOP: 5–21 mmHg or preop. IOP↓ ≥ 20% No need of postop. carboanhidrase inhibitors No need of reoperation **Success rate (%):** M1: 72.7 M4: 54 M6: 41 last: 27.3	*Achieved IOP ± SD (mmHg) and* *preop. IOP↓ (%):* 16.7 ± 4.58 *(p < 0.028)* •No complications
Williams et al. [29], 2018 Retrospective	**Number of eyes (number of patients):** 79 (79) **Etiology (number of eyes):** POAG (40) CACG (18) Pseudoexfoliative glaucoma (9) Uveitis-associated glaucoma (3) Neovascular glaucoma (6) Pigmentary glaucoma (3) **Follow-up (months ± SD):** 7.8 ± 4.5 **Preop. IOP (mmHg):** 31.9	**Definition of success (IOP, lowering):** IOP: 6–21 mmHg or preop. IOP↓ ≥ 20% **Success rate (%):** M3: 75 M6: 66 last: 67	*Achieved IOP ± SD (mmHg) and preop. IOP↓ (%):* end of follow-up: 31.9 ± 10.2; 51% •Complications: hypotony (7) persisting inflammation (21) decrease of BCVA (13) macular edema (4) corneal edema (2) phthisis (2)
Yelenskiy et al. [30], 2018 Retrospective	**Number of eyes (number of patients):** 161 (197) **Etiology (number of eyes):** POAG (141) Neovascular glaucoma (8) Uveitis-associated glaucoma (4) CACG (4) Post-PKP glaucoma (3) ICE syndrome (1) **Follow-up (months ± SD):** 12 **Preop. IOP (mmHg):** 22	**Definition of success (IOP, lowering):** IOP: 6–18 mmHg or preop. IOP↓ ≥ 20% **Success rate (%):** 71	*Achieved IOP (mmHg) to the end of follow-up:* 15.8 *(p < 0.001)* •Anti-glaucoma medications decreased from 3 to 2. •Complications: 4 patients: macular edema
Emanuel et al. [31], 2017 Retrospective	**Number of eyes (number of patients):** 84 **Etiology (number of eyes):** POAG (49) CACG (6) Pseudoexfoliative glaucoma (8) Uveitis/Inflammation-associated glaucoma (2) Mixed mechanism glaucoma (2) Post-traumatic glaucoma (2) Pigment dispersion glaucoma (1) Unknown origin (1) **Follow-up (months ± SD):** 4.3 ± 3.0 **Preop. IOP (mmHg):** 27.7	**Definition of success (IOP, lowering):** n/a **Success rate (%):** n/a	*Achieved IOP ± SD (mmHg) and preop IOP↓ (%):* M1 16.3 ± 9.5; 41 M3: 14.6 ± 8.8 M6: 13.0 ± 6.9 M12: 11.1 ± 4.4 •Complications: hypotony (11) 46%: persisting inflammation 41% decrease of BCVA
Lee et al. [32], 2017 Retrospective	**Number of eyes (number of patients):** 36 (34) adult: 27 (25); pediatric: 9 (9) **Etiology (number of eyes):** adult: POAG (12) Steroid-associated glaucoma (5) Neovascular glaucoma (4) Post-PKP glaucoma (2) Congenital glaucoma (2) Post-traumatic glaucoma (1) Aphakia (1) pediatric: Sturge-Weber sy. (4) Aphakia (2) Peter’s anomaly (1) PHPV (1) Congenital glaucoma (1) **Follow-up (months):** 12 **Preop. IOP (mmHg):** adult: 28.41; pediatric: 34.28	**Definition of success (IOP, lowering):** (1) IOP: 6–21 mmHg or preop. IOP↓ ≥ 20% (2) no use of oral CAI (3) no loss of light perception vision (4) no reoperation within 12-month follow-up period **Success rate (%):** adult: 72.22 pediatric: 22.22	adult: *Achieved IOP ± SD (mmHg):* M1: 14.44 ± 6.38 M3: 18.56 ± 7.66 M6: 18.62 ± 6.64 M12: 18.98 ± 6.45 •3 patients underwent additional glaucoma surgery pediatric: *Achieved IOP ± SD (mmHg):* M1: 20.44 ± 13.41 M3: 23.56 ± 10.10 M6: 23.00 ± 8.31 M12: 27.20 ± 15.68 •7 patients required reoperation •No complications
Kuchar et al. [33], 2016 Retrospective	**Number of eyes (number of patients):** 19 **Etiology (number of eyes):** Secondary open angle glaucoma (6) POAG (5) PACG (5) Neovascular glaucoma (3) **Follow-up (months):** 2 **Preop. IOP (mmHg):** 37.9	**Definition of success (IOP, lowering):** IOP: 6–21 mmHg or preop. IOP↓ ≥ 20% (with/without anti-glaucoma medications) **Success rate (%):** 73.7	•*Preop. IOP↓ (%):* end of follow-up: 40,1% •Number of anti-glaucoma medications decreased from 2.6 to 1.9. •Complications: decrease of BCVA (4) hypotony (1) •Repeated treatments: 3 patients (15.8%) 1 time
Aquino et al. [34], 2015 Prospective, randomized	**Number of eyes (number of patients):** 14–14 (28) **Etiology (number of eyes):** MP-TSCPC/ CW-TSCPC: POAG (5/6) PACG (5/1) Neovascular glaucoma (7/12) Other glaucoma (7/5): Silicon oil induced glaucoma Aphakia Post-traumatic glaucoma **Follow-up (months ± SD):** 17.5 ± 1.6 **Preop. IOP (mmHg):** 36.5	**Definition of success (IOP, lowering):** IOP: 6–21 mmHg or preop. IOP↓ ≥ 30% (with/without anti-glaucoma medications) **Success rate (%):** MP-TSCPC: M12: 75; M18: 52; *(p < 0.01)* CW-TSCPC: M12: 29; M18: 30; *(p < 0.13)*	•Number of anti-glaucoma medications decreased: Group MP-TSCPC: from 1.75 to 1.00, Group CW-TSCPC: from 3.00 to 2.00 •Complications: loss of vision (1) persisting inflammation (1) •Repeated treatment: 30% of patients 1 time; 17% of patients 2 times.
Tan et al. [35], 2010 Prospective	**Number of eyes (number of patients):** 40 (38) **Etiology (number of eyes):** Neovascular glaucoma (16) PACG (10) POAG (9) Aphakia (2) Silicon oil induced glaucoma (2) Juvenile glaucoma (1) **Follow-up (months ± SD):** 17.3 ± 2.0 **Preop. IOP (mmHg):** 40.1	**Definition of success (IOP, lowering):** IOP: 6–21 mmHg or preop. IOP↓ ≥ 30% (with/without anti-glaucoma medications) **Success rate (%):** 72.7	*Achieved IOP ± SD (mmHg):* D1: 31.1 ± 13.4 M1: 27.4 ± 12.7 M6: 25.8 ± 14.5 M12: 24.7 ± 10.8 end of follow-up: 24.6 ± 9.6 •Number of anti-glaucoma medications decreased from 0.72 to 0.15 at M12. •Complications: persisting inflammation (4) hyphaema (7)

IOP↓: intraocular pressure decrease.
